# The role of gender-specific factors in the choice of specialty training in obstetrics and gynecology: results from a survey among medical students in Germany

**DOI:** 10.1007/s00404-021-06232-2

**Published:** 2021-09-22

**Authors:** Maximilian Riedel, André Hennigs, Anna Maria Dobberkau, Caroline Riedel, Till Johannes Bugaj, Christoph Nikendei, Niklas Amann, Anne Karge, Gabriel Eisenkolb, Maria Tensil, Florian Recker, Fabian Riedel

**Affiliations:** 1 Department of Gynecology and Obstetrics, Klinikum rechts der Isar, Technical University of Munich, Munich, Germany; 2 Department of Gynecology and Obstetrics, Heidelberg University Hospital, Heidelberg, Germany; 3 Department of General Internal Medicine and Psychosomatics, Heidelberg University Hospital, Heidelberg, Germany; 4 Department of Obstetrics and Gynecology, Ludwig Maximilians University (LMU), Munich, Germany; 5 Kirinus Clinic for Psychotherapy, Munich, Germany; 6 Department of Obstetrics and Gynecology, Bonn University Hospital, Bonn, Germany

**Keywords:** Gender, Medical teaching, Specialty training, Obstetrics and gynecology, Students

## Abstract

**Purpose:**

The field of obstetrics and gynecology (OB/GYN) is facing growing competition for young professionals in Germany, with high interest rates among female graduates and a declining proportion of male students who choose residency training in the field. The aim of this study is to analyze general and gender-dependent factors that influence the decision for or against specialty training in OB/GYN among medical students in Germany.

**Methods:**

Between February and November 2019, *n* = 346 medical students in their 5th and 6th year of undergraduate training at Heidelberg University received a questionnaire with 44 items.

**Results:**

*n* = 286 students (61.3 female; 38.7% male) participated in the study. 28% of the female students and 9% of the male students had considered OB/GYN for their specialty training. The students reported different general and gender-specific influencing factors in their choice of a specialty. Both genders desired a good work-life-balance, however, in comparison with their female colleagues, male students had heavily weighted factors related to their later careers and professional success, including competition among colleagues. Male students had gained little practical experience during compulsory internships (26.9% for females vs. 8.8% for males) or had chosen their final-year elective in OB/GYN (15.9% for females vs. 5.5% for males). Female students had worried about the negative effects of their sex on their career (35.4% for females vs. 5.9% for males).

**Conclusion:**

OB/GYN must become more appealing and attractive to young female and male professionals alike. A better compatibility of career and family should go hand in hand with the implementation of differentiated, (extra) curricular teaching approaches that take the different preferences of female and male students into account.

## Introduction

The development of improved gender diversity among medical students and professionals has considerably accelerated throughout the world in recent decades. In the USA, women comprised the majority of students (50.5%) at American medical colleges in 2019 for the first time [1]. This trend has also been seen in the field of obstetrics and gynecology (OB/GYN) [2]. Similarly, in Germany, the gender distribution of medical students and physicians has shifted considerably. First, the share of female students in medicine has increased continuously, with women comprising two-thirds of all medical students today [3]. From a total of 98,763 medical students in Germany, 61,000 (62%) were female in the 2018/19 winter semester [4]. Second, gender-dependent preferences have changed for some specialties yet remained stable for others during the same period of time. In OB/GYN, more than 80% of residents (Assistenzarzt/-ärztin) are female, while the majority of residents in other “classical” surgical specialties (e.g., general surgery, neuro-surgery, orthopedics) are male [5–7]. The share of male physicians who complete specialist training (Facharzt/-ärztin) in OB/GYN has shown a downward trend over the last three decades. In 2019, 93 (13.6%) male vs. 598 (86.4%) female physicians finished their specialist training in OB/GYN in Germany [7].

Women’s preference for the specialty at the beginning of their careers stands in contrast to the distribution of leadership positions in OB/GYN as female physicians reach these positions less frequently compared with their male colleagues. Women are underrepresented among senior physicians (Oberarzt/-ärztin) and clinical directors (Chefarzt/-ärztin) [8]. At the same time, the lack of physicians in outpatient and inpatient medical care will continue to grow in the coming decades [9]. The total number of physicians who finish specialty training in OB/GYN has stagnated at around 700 per year [7], with an over-proportionate number of female physicians working part-time [10]. Recruiting sufficient personnel for senior positions in OB/GYN has become an increasingly challenging task [11].

Understanding exactly what factors contribute to the final choice of a specialty remains an ongoing research question in the international literature. Few data dealing with gender-dependent factors in the choice for or against a clinical career in OB/GYN have thus far been collected in Germany. Apart from general career goals, such as motivation, practical experience, and maintaining a healthy work–life balance, the present study also focuses on gender-specific differences in students’ perceptions of OB/GYN that may influence their choice of a specialty.

## Methods

### Participation and criteria for inclusion

Medical students in their 4th or 5th year of study before the beginning of their final year were invited to participate in the study. All participating students were enrolled at the Faculty of Medicine at Heidelberg University, Germany. The participants came from two groups. First, students who had participated in the four-week module “obstetrics and gynecology” within the compulsory curriculum of their medical studies were asked to participate (*n* = 176 out of 286; 61.5%). In addition, the department of obstetrics and gynecology at Heidelberg University Hospital offers a voluntary 2-day extracurricular preparation course for the standardized written exam before the beginning of the final year. Further participants were recruited from this course (*n* = 160 out of 286; 38.5%). The study took place between February and November 2019. Participation was entirely voluntary, and non-participation had no consequences for a student’s further course of study.

### Context of medical studies in Germany at the time of the study

Medical studies in Germany are divided into three phases. The first two, pre-clinical years (Vorklinik) deal for the most part with basic science (anatomy, physiology, biology, chemistry, physics, etc.) in preparation for the following three years of comprehensive clinical teaching (Klinik) [12]. The clinical curriculum comprises in-depth teaching of all major and minor specialties in modules including lectures, seminars, and bedside teaching. The final year (Praktisches Jahr, PJ) concludes the medical studies [13]. During this final year, students work full-time in three blocks of 16-week training courses in internal medicine, surgery, and an elective specialty of their choice. Unlike in the United States or the United Kingdom, there is no standardized or federal residency program for medicine in Germany. Students who aspire to complete residency apply directly with the head of the department or outpatient clinic. The final year of medical studies is therefore particularly important for medical students as it represents the transition toward their later occupation.

### Informed consent

An ethics approval was obtained in advance from the Ethics Committee of Heidelberg University (S-211/2019).

### Study design

Students were asked about their motivations and reasons for or against their choice of a specialty both in general and for OB/GYN, in particular, via an anonymous questionnaire. The evaluation of gender-specific factors in choosing specialty training was particularly relevant. The questionnaire was distributed at the end of the aforementioned courses. It included a total of 44 items, 31 of which were rated on 5-point Likert scales [1 = “strongly disagree (–)”, 2 = “disagree (−)”, 3 = “neither agree nor disagree (−/ +)”, 4 = “agree” ( +), 5 = “strongly agree” (+ +)], while the others were either dichotomous or classification questions.

### Statistical analysis

The assessment was conducted via R^®^ (version 4.0.3, R Core Team). Tables and figures were generated in Word^®^ (version 2019, Microsoft) and GraphPad Prism^®^ (version 9.0.1, GraphPad Software). *p* values for statistical significance between female and male students were calculated using unpaired *t* tests. *p* values < 0.05 were defined as statistically significant. Mean and relative values were calculated descriptively for each individual item. Possible gender-dependent interactions were calculated using a generalized linear model (GLM) with de-sparsified lasso.

## Results

### General and gender-specific factors in the choice of a specialty

*n* = 286 students (female: 61.3; male: 38.7%; participation rate: 82%) completed the study. Interest in the subject matter, the subject-specific organs, or pathologies as well as a good working atmosphere were critical factors for the majority of both female and male students when choosing a specialty. By contrast, career factors—such as good career opportunities or potential earnings —were less important. Both the female and male students strived to maintain a decent work–life balance, but the women more often sought to establish their own practice later (Table 1). In addition, a significant difference in the Likert scale between female and male students with regard to the relevance of career attributes was found. Attributes, such as scientific work, competition among colleagues, and status/prestige, were more appreciated by male students (Table 2). Female and male students considered the same experiences before and during their studies to be relevant to their personal choice of a specialty. The strongest factors in this context were positive experiences during internships, the final-year role models/mentors in the clinical setting, and the quality of the medical teaching, whereas experience with nursing abroad and influence from relatives or friends were less important (Table 3). Male students tended to consider conducting research and writing a thesis for a medical doctoral degree to be more influential on their decision (female: 19%; male: 30%; *p* value: 0.05). Female students worried significantly more than male students about not finding mentors or role models of the same sex (mean: 1.4 vs. 2.1; *p* value: < 0.001) and that their sex could be associated with disadvantages later in their clinical careers (mean: 1.4 vs. 2.8; *p* value: < 0.001).

**Table 1:** Internal and external factors that positively influence the choice of a specialty

	Female students		Male students		*p* value
	*N*	%	*n*	%	
Working hours, working atmosphere, colleagues	130	74	80	72	0.741
Interest in the subject matter and procedures	127	72	79	71	0.857
Compatibility of family and career	134	76	73	66	0.063
Interest in subject-specific organs and pathologies	117	66	61	64	0.665
Opportunity to establish oneself in practice	113	64	58	52	**0.047**
Career opportunities	42	24	32	29	0.358
Potential earnings	26	15	22	20	0.279

Students’ responses (in absolute and relative numbers) to the following statement: “what factors positively influenced your choice of a specialty?”

Statistically significant answers are depicted in bold

**Table 2:** Factors that positively influence the choice of a specialty

	Likert scale (mean)		*p* value
	Female	Male	
Collegiality and a good working atmosphere	4.6	4.4	0.089
Wide range of subject matter	4.0	3.9	0.283
Even gender distribution among colleagues	3.8	3.6	0.099
Manual work and surgery	3.6	3.4	0.211
Emergency situations	3.4	3.6	0.266
Night and shift work	3.2	2.9	0.088
Scientific work	2.6	2.9	**0.045**
Accepting greater workloads and career competition	2.4	2.7	**0.022**
Status and prestige of the specialty	1.9	2.3	**0.0016**

Mean Likert scale (“1” = completely disagree; “5” = completely agree) depicting factors that positively influence the choice of a specialty

Statistically significant answers are depicted in bold

**Table 3:** Experiences that positively influence the choice of specialty training

	Female students		Male students		*p* value
	*n*	%	*n*	%	
Practical year (PJ)/ internship (Famulatur)	140	80	81	73	0.209
Physicians as role models/mentors in the clinic	130	74	82	74	0.998
Quality of medical teaching	106	60	56	50	0.107
Science/doctoral thesis	34	19	33	30	0.050
Part-time employment/vocational training	40	23	22	20	0.557
Friends, relatives, etc	43	24	24	20	0.582
Nursing placement	31	18	11	10	0.058
International studies (e.g. Erasmus)	22	12	11	11	0.663

Students’ responses (in absolute and relative numbers) to the following statement: “What experiences positively influenced your choice of specialty training?”

### Gender-dependent perception and experience in obstetrics and gynecology

Female and male participants had equally decided on a specialty at the time of the study (mean: 3.5 vs. 3.3; *p* value: 0.15). Significantly more female (28%) compared with male (8%) students had considered a future career in OB/GYN. In addition, internal medicine, surgery, and pediatrics were among the most-preferred specialties, independent of gender (Table 4). Both female and male students disagreed with the statement that women’s healthcare should rest solely in the hands of female physicians (mean: 1.5 vs. 1.6; *p* value: 0.35).

**Table 4:** Students’ preference for specialty training

	Female students		Male students		*p* value
	*n*	%	*n*	%	
Obstetrics and gynecology	63	28	12	8	** < 0.001**
Internal medicine	33	15	32	21	0.055
Pediatrics	35	15	15	10	0.152
Surgery	19	8	14	9	0.645
General medicine	12	5	7	5	0.864
Orthopedics	11	5	4	3	0.300
Anesthesiology	7	3	12	8	**0.040**
Neurology	8	4	6	4	0.747
Radiology	2	1	4	3	0.207
Radiation therapy	4	2	3	2	0.822
Psychiatry and psychotherapy	3	1	6	2	0.120
ENT (ear, nose, throat)	3	1	6	4	0.120
Ophthalmology	4	2	1	1	0.342
Dermatology	2	1	7	5	**0.037**
Urology	4	2	5	3	0.328
Pathology	0	0	0	0	–
Other	17	7	16	11	0.239

Statistically significant answers are depicted in bold

Compared with male students, their female counterparts had gained greater practical experience in OB/GYN during their studies via nursing placements (17.8 vs. 7.7% vs; *p* value: < 0.001), internships (26.9 vs. 8.8%; *p* value: < 0.001), and (planned) electives in the final year of study (15.9 vs 5.5%; *p* value: < 0.001). A lack of interest in the subject matter had more often deterred male students from the specialty (15.9 vs. 5.5%; *p* value: 0.0022). By contrast, more female students had considered the heavy work- and shift load negatively (25 vs. 10.3%; *p* value: 0.0023). The intimacy of the medical topics (4.6 vs. 6.9%; *p* value: 0.243), the treatment of exclusively female patients (22.2 vs. 25.9%; *p* value: 0.331), and the high share of operative medicine in OB/GYN (22.2 vs. 17.2%; *p* value: 0.418) were found to be gender-independent (females vs. male) reasons for not pursuing a career in the field.

Next, the requirements of the fields of gynecology, (general) surgery, and urology were investigated in a comparative analysis. The students were asked to assess the share of the workload in the three areas of operative medicine, conservative oncology, and emergency medicine within the three fields. The gradual Likert scale revealed that the share of the workloads for the three areas within gynecology and urology was evaluated as being quite balanced, whereas (general) surgery was evaluated as having a clear predominance of operative medicine. No gender-dependent differences were observed except for assessments of the role of emergency medicine in gynecology (*p *value: 0.036) (Table 5).

**Table 5:** Students’ differential perceptions of gynecology, urology, and surgery

	Likert scale (mean)		*p* value
	Female	Male	
The share of the workload of operative medicine is high in			
Gynecology	3.5	3.4	0.195
Urology	3.5	3.5	0.829
(General) surgery	4.5	4.5	0.516
The share of the workload of conservative oncology is high in			
Gynecology	3.8	3.6	0.059
Urology	3.3	3.3	0.634
(General) surgery	2.6	2.9	0.080
The share of the workload of emergency medicine is high in			
Gynecology	3.8	3.5	**0.036**
Urology	3.0	3.0	0.778
(General) surgery	4.3	4.10	0.266

Mean Likert scale (“1” = completely disagree; “5” = completely agree) depicting students’ differential perception of gynecology, urology, and surgery

Statistically significant answers are depicted in bold

### Gender-dependent differences in the perception of and experience with the gynecological examination

Independent of gender, the majority of both female and male students considered the basic gynecological examination to be an important part of their clinical training (mean female: 3.8; mean male: 3.6; *p* value: 0.18) and stressed that all practicing physicians must be qualified to perform the examination (mean female: 3.6; mean male: 3.7; *p* value: 0.81). This finding contrasts with the practical experience of male students. Compared with their female counterparts, male students had less frequently observed (30 vs. 47%) or carried out (8 vs. 24%) an examination (under supervision). In total, 62% of male students versus 29% of female students had neither observed nor conducted a gynecological examination on their own during their studies (*p* value: < 0.001).

### Gender-dependent preferences in the choice of a sub-specialization

**Fig. 1:**
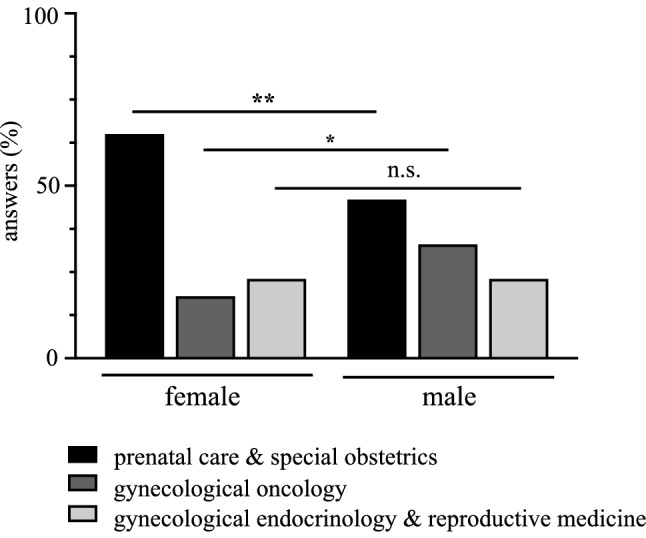
Bar diagram depicting female (*n* = 176) and male (*n* = 110) preferences (in %) for one of the sub-specializations in OB/GYN (pre-natal care and special obstetrics, gynecological oncology, and gynecological endocrinology and reproductive medicine) in different gray scales

After completing specialist medical training in OB/GYN, sub-specializations in pre-natal care and special obstetrics, gynecological oncology, and gynecological endocrinology and reproductive medicine are possible. In terms of the preferences for these sub-specializations among our students, a high preference level—especially among female students—was reported for pre-natal care and special obstetrics (female: 65%; male: 46%; *p* value: 0.002). Male students, on the contrary, opted significantly more often for a sub-specialization in gynecological oncology (female: 18%; male: 33%; *p* value: 0.005). Students showed balanced interest in gynecological endocrinology and reproductive medicine, independent of their gender (female: 23%; male: 23%; *p* value: 0.829) (Fig. 1).

## Discussion

Our data shed light on the different perceptions and experiences of female and male students in OB/GYN during their medical studies. These differences correspond to the later highly gender-dependent choice of specialty training in OB/GYN. At the time of this study, most participants were ready to begin their final year of study and were therefore approaching the decision for or against a certain type of specialty training. Our data support the findings of previous studies as the students in our cohort (female: 58%; male: 48%) were highly sure of their choice of specialty training. About one-fourth (female: 24%; male: 26%) of students had not yet reached a decision.

Deciding on a certain type of specialty training is a very complex and dynamic process. Some students had made their decision very early during their studies, while others had made or planned to make the final decision just before, during, or even after their final year. Decision-making depends on both internal and external factors. Internal factors include gender, socio-economic background, individual life and family planning, expected future income, and interest or talent. External factors, by contrast, include experiences during internships or electives as well as influence by family members, friends, mentors, or role models in the clinical setting [14–16]. Our study reveals that career opportunities, potential earnings and the opportunity to do scientific work—that is in the setting of a university hospital also a relevant career factor—are of minor importance for our students. This corresponds to the international literature as, for example, demonstrated in two US publications. Among 205 first- and second-year medical students, income prospects and prestige within the medical profession were only weak and gender-independent factors for choosing OB/GYN in their career [17]. In another qualitative survey, income was likewise of varying and mostly low importance to students [18]. Similar to our results, in a cohort of 465 German medical students, work–life balance was the most important factor for choosing an employer and for general satisfaction with the job [19, 20]. However, the individual expectation to actually have a good work–life balance in the job was the lowest among students that plan to pursue a career in OB/GYN. These negative expectations also aggravated from the beginning to more advanced stages of the medical studies [21]. Moreover, an American study including 1.327 students reported OB/GYN to be the most “lifestyle-unfriendly” among 18 other specialties [22]. These investigations underline that structural changes in the clinical setting that support the compatibility of career and family are necessary, in particular, with regard to potential shortage of physicians in OB/GYN in future.

Compared with other specialties with a high operative workload, studies in OB/GYN are particularly prone to deterring potential training candidates between the beginning of the clinical stage in the third year and the final year [23]. External factors—such as positive experiences with internships, electives, or medical classes—have been found to play a decisive role in final career decisions, and these practical experiences were particularly relevant for the students in our study. In general, positive experiences can be seen to be an important motivational factor for students when choosing to engage in a specialty in greater depth and eventually selecting it for later training [24]. As already demonstrated, extracurricular preparation courses for the state exam have a potentially positive impact on the decision of specialty training in OB/GYN [25, 26].

Studies indicate that male students have less practical experience in OB/GYN compared with female students [27]. Our data reveal that 78% of male students had no practical experience beyond the compulsory albeit limited bedside teaching in the OB/GYN course. One American publication reported that male students had been dissatisfied with the practical experience in OB/GYN and suggested that this was due to their gender [28]. The male students in the aforementioned study had fewer opportunities to actually learn and practice the gynecological examination [28, 29], which is consistent with the finding of our study as 62% of our male students had neither observed nor conducted an examination on their own. However, this finding contrasts with the gender-independent majority view in our study—namely that all physicians should be competent in gynecological examinations and that this skill should be part of the compulsory curriculum for medical teaching. The literature reveals that the discrepancy between these practical experiences is not due to patients’ primary preference for female examiners because satisfaction with the examination does not correlate with the sex of the examiner [30, 31]. Empathy and good communication skills are crucial in this context [32]. By contrast, evidence suggests that supervisors are in fact the strongest obstacle to learning the gynecological examination as they can actively prohibit male students from participating or passively exclude them [33].

Gender-dependent issues and challenges exist in both medical studies and specialty training. We observed that female students, in particular, expect disadvantages in their later career due to their sex. This finding is consistent with reports from international studies indicating that female students experience more gender discrimination and less support from mentors and that they are less confident in their academic achievements in OB/GYN [34]. In general, this problem applies more frequently to women; however, in one American study, men and women alike reported the highest degree of discrimination in (general) surgery and OB/GYN [35]. The authors speculate that a variety of reasons—such as patient characteristics, daily work, interaction with nursing staff, or the sex of supervisors or colleagues—might account for this finding [35]. Interestingly, these two specialties are also among those with the highest levels of gender discrepancy among physicians in Germany [7]. Therefore, a well-directed and active promotion of women in their clinical and research career is needed that also respects and supports women in their wish to have children. Simultaneously, potential barriers in the clinical and academic setting have to be torn down that make male students feel uncomfortable or not fitting for the specialty.

Several international studies have investigated the role of gender on the choice of specialty training. The heterogeneous results indicate that this decision also depends on cultural and socio-economics factors. While Swedish medical students, for example, have gender-independent preferences [36], female students in Saudi Arabia more frequently pursue a career in surgery, ophthalmology, or pediatrics [37]. In general, the global trend is for female students to favor general medicine, pediatrics, or OB/GYN [35]. These findings are consistent with the data from our study. Moreover, in our study, male students showed little interest in OB/GYN. This finding corresponds well with those of the international literature, although the high reported preference levels of male students for other specialties—usually those with a demanding surgical workload, such as (general) surgery [38], orthopedics [39], neurosurgery [40], or urology [41]—were not found in our analysis.

Factors that explicitly distinguish OB/GYN from other specialties—such as the exclusively female patients or the intimate nature of the female medical problems—did not play a distinguishing role in the choice of female vs. male students for specialization in the field. Comparing OB/GYN with (general) surgery, the operative workload was less significantly considered, independent of gender. Interestingly, the opportunity to work surgically was reported by one American study as being a crucial factor in male students’ interest in OB/GYN [17]. We speculate that this could be one reason why more male students who want to pursue primarily surgical training might be put off by OB/GYN due to the broad spectrum and diverse sub-specializations of the field [42]. We suggest that an improved and more-diversified public portrayal of the specialty with regard to the high and challenging operative workload may attract students that want to pursue a surgical career, however, do not consider OB/GYN fitting to their occupational aspirations at first. Also, extracurricular activities and courses for students that stress unique sub-specializations and gender-specific preferences, for example in gynecological surgery or prenatal care, could be reasonable approaches for a better promotion of OB/GYN. This has been shown to be successful in the case of neurosurgery [43] or general surgery [44].

### Limitations

To the best of our knowledge, this is the first survey among German medical students to provide an in-depth analysis of the gender-specific elements that influence the choice for or against specialty training in OB/GYN. Limitations in our results stem from the monocentric approach at Heidelberg University and the use of a questionnaire to collect data. Further research approaches in the future may use additional qualitative methods (for example, structured interviews). Our cohort of students formed a rather homogeneous group as all of them were in an advanced phase of their studies, and we could therefore not investigate dynamic processes with regard to gender-dependent preferences over the entire course of study. However, it was advantageous for our study to focus on students who needed to choose their specialty training in the near future as these students may have already deliberately given thought to the choice. Students filled out the questionnaire after either the OB/GYN curricular module or the state-exam-preparation course in OB/GYN. Therefore, a response bias from the very recent theoretical and practical experience in the specialty—either positive or negative—cannot be excluded. Although the questionnaires were answered anonymously and individually, we could not exclude an effect caused by social desirability, for example, for items dealing with gender equality or the work–life balance.

## Conclusion

Competition among young professionals has been intensifying in Germany in recent decades. This competition is particularly relevant in OB/GYN as the field now faces two simultaneous issues: first, female physicians are underrepresented in leading clinical and scientific positions, and second, specialty training in OB/GYN has become increasingly less appealing for male students in the last three decades. It is therefore now necessary to take actions to improve the recruitment of male and female physicians alike. First, for both gender, the reconciliation of career and family is of major importance outweighing financial incentives by far. Limiting the number of night shifts and ensuring predictable working hours are especially relevant for OB/GYN due to the nature of the delivery room and its frequent need to work late-night. Second, the lack of interest in OB/GYN among male students goes along with little practical and hands-on experience in the specialty. We propose that specific extra-curricular and practical courses could fill this gap and attract students that have not considered OB/GYN for their career yet, for example, due to a misconception of the relevance of the surgical workload. New approaches in this regard are illustrated in the new information and promotion portal for medical students “Gyn werden” hosted by the *Young Forum of the German Society for Obstetrics and Gynecology* (Junges Forum in der DGGG) [45]. This website aims to motivate female and male students alike to choose specialty training in OB/GYN in Germany. We also believe that the conclusions reached in this study should also applicable for other healthcare systems around the world.

## Data Availability

All (raw) data and material are available upon reasonable request.
